# Application of pedicled greater omentum flap tamponade combined with laparoscopic fenestration in diaphragmatic hepatic cyst

**DOI:** 10.1186/s12893-022-01807-5

**Published:** 2022-10-29

**Authors:** Zujian Wu, Yun Chen, Ye Jin, Changfeng Liu, Yusu Liu, Bing Zhang

**Affiliations:** 1 General surgery, Tongde Hospital of Zhejiang Province, 310012 Hangzhou，Zhejiang Province, China; 2 Hepatobiliary and Pancreatic Surgery, Tongde Hospital of Zhejiang Province, 310012 Hangzhou, Zhejiang Province China; 3 Second Clinical Medical College, Zhejiang Chinese Medicine University, 310012 Hangzhou, China

**Keywords:** Laparoscopic fenestration, Diaphragmatic hepatic cyst, Vascular pedicled greater omental flap

## Abstract

**Objective:**

To investigate the efficacy and clinical application advantage of omental tamponade with vascular pedicle combined with Laparoscopic fenestration for the treatment of diaphragmatic hepatic cyst.

**Methods:**

A total of 56 patients with diaphragmatic hepatic cysts underwent laparoscopic surgery in a single tertiary academic medical center from January 2010 to October 2020, including 21 patients (non-omental group) underwent laparoscopic fenestration of liver cysts, and 36 patients underwent laparoscopic liver cyst fenestration combined with vascular pedicle omentum tamponade (omental group). The general conditions and follow-up results of the two groups were compared and annalyzed.

**Results:**

The operation time of the omental group was longer than that of the non-omental group (P = 1.358E-4). There was no significant difference in postoperative complications, postoperative laboratory values and hospital costs (P>0.05). The length of hospital stay in omental group was shorter than that in non-omental group (P = 0.034). In the omental group, recurrence occurred in 1 of 35 patients (4.65%) who were followeded up 12 months after surgery. In the non-omental group, of the 21 patients followed, 3 patients (14.28%) recurred 6 months after surgery, and 8 patients (38.10%) recurred 12 months after surgery.

**Conclusion:**

It is an effective method to prevent the recurrence of diaphragmatic hepatic cyst after laparoscopic fenestration by packing the cyst with vascularized omentum.

**Supplementary Information:**

The online version contains supplementary material available at 10.1186/s12893-022-01807-5.

## Introduction

Simple liver cyst (SLC) is considered to be the most common disease in the liver. Its prevalence is 2.5–5% of the general population. It has a high incidence in women (male to female ratio of about 1 to 4). About 5% of patients may develop symptoms and require surgical intervention [1, 2]. In addition to the tricky treatment of some complex polycystic liver disease, which requires lobectomy and even liver transplantation [3], laparoscopic fenestration and drainage has become the gold standard for the treatment of symptomatic SLC [4, 5]. However, for liver cysts located on the diaphragm surface, especially those near the diaphragm surface in Couinaud IV, VI, and VIII segments, the diaphragm may easy to cover and close the opening window after the liver cyst fenestration, resulting in postoperative cyst recurrence [6, 7].

The greater omentum is a highly expandable structure. After fusion with the injured tissue, the activated omentum can secrete a large number of powerful growth factors (VEGF, bFGF, etc.), progenitor cells and chemokines (such as SDF-1α) to vascularize, debride, stop bleeding, heal and regenerate the tissue [8, 9]. The pedicled greater omentum flap is prepared by detaching from the greater curvature of the stomach and preserving the gastroepiploic vessels to keep the blood supply of the flap intact. The advantage of this technique is that the flap can be moved away from the greater omentum, thereby reaching the chest, pelvis, and bottom of the trunk where it cannot [10]. Based on these special biological characteristics of the greater omentum, it has been successfully used in general surgery, neurosurgery, cardiothoracic surgery, urological surgery, gynecological surgery and other surgical operations [10–15].

The purpose of this single-center study is to compare the efficacy of laparoscopic deroofing combined with vascular pedicled greater omental flap tamponade and simple laparoscopic fenestration in diaphragmatic hepatic cyst patients.

## Materials and methods

### General information collection

Retrospectively analyzed the patients who underwent laparoscopic diaphragmatic hepatic cyst surgery in Tongde Hospital of Zhejiang Province from January 2010 to October 2020. Collect information on demographics, clinical manifestations, liver function laboratory indicators, radiography, surgical interventions, and pathology of the final specimens of all patients. All patients had a preoperative ultrasonography (US) and computed tomography (CT) scan of the abdomen. If the US or CT image showed features suggestive of cystadenoma or cystadenocarcinoma, further examination of magnetic resonance imaging (MRI) was made. The patients were divided into two groups, they are simple laparoscopic fenestration group (non-omental group) and laparoscopic fenestration combined with omental tamponade group (omental group). The research proposal was approved by the Human Ethics Review Committee of Tongde Hospital of Zhejiang Province.

### Surgical methods

#### General Operation

All patients were under general anesthesia with endotracheal intubation. The patient’s head was elevated 20 ° to 30 ° and tilted left or right to facilitate exposure and operation. A 10 mm long incision was made at the upper edge of the umbilicus to establish a pneumoperitoneum with a pressure of 12 to 14 mmHg, a 10 mm trocar was inserted, and the laparoscope was placed to observe the situation in the abdominal cavity and examine the location, number, size of the cysts and adhesion in detail. A 10 mm trocar was placed below the xiphoid process, a 5 mm trocar below the right costal margin, in patients with hepatic cysts on the left side of the falciform ligament, another 5 mm trocar below the left costal margin was added.

#### Fenestration of hepatic cyst

**Fig. 1:**
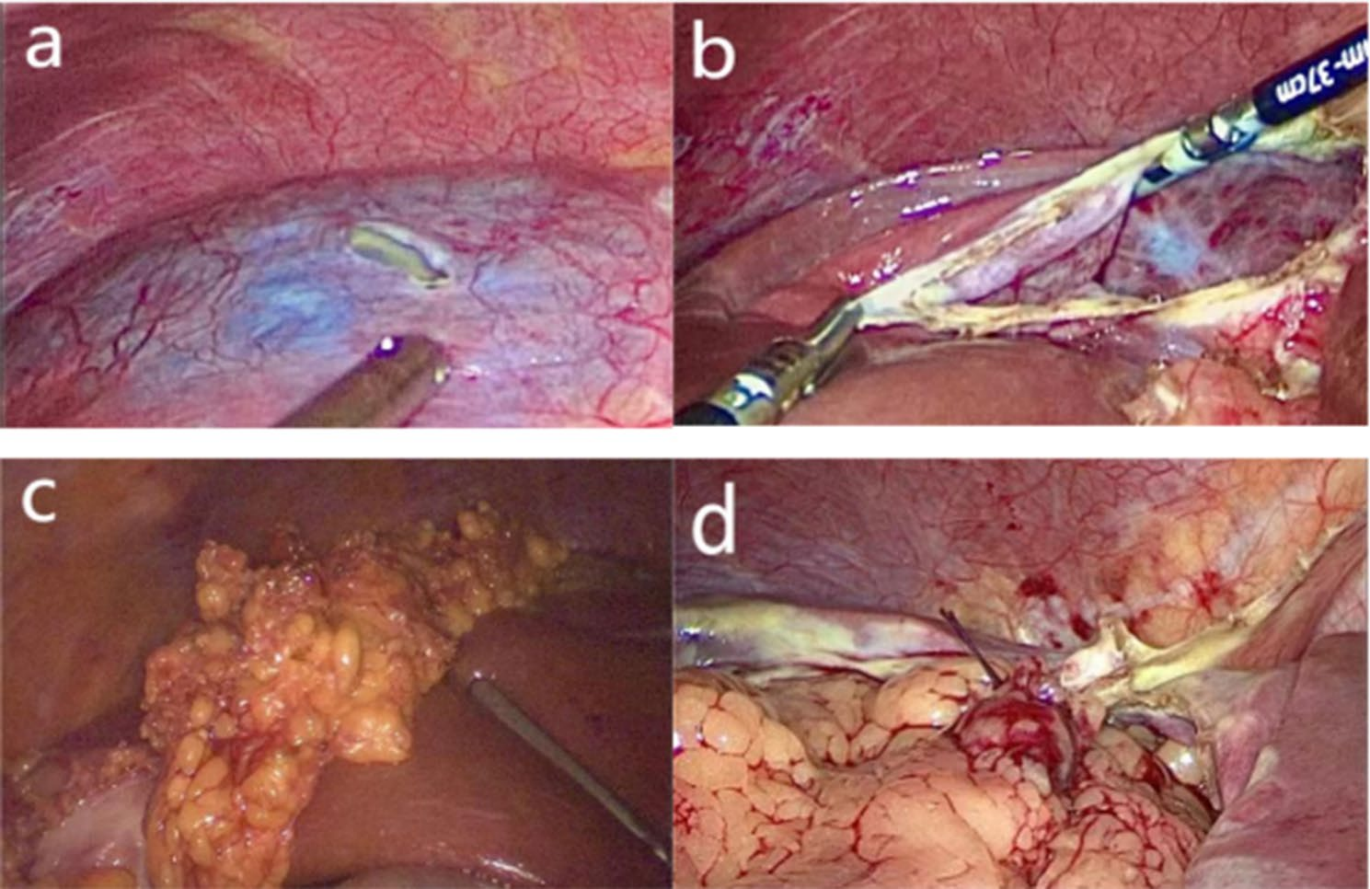
Operative procedure **a** The hepatic cyst on the diaphragmatic surface was incised and the effusion in the hepatic cyst was fully aspirated. **b** The cyst wall was resected as far as possible to reach more than 1/2 of the diameter of the cyst, and the septa in the hepatic cyst were completely separated. **C** Pedicled omental flap was dissected and 1?2 vessel were preserved, and sufficient extension of the omental flap was maintained. **d** The greater omentum flap with pedicle should be used to fully fill the hepatic cyst cavity, and more than 80% of the cyst cavity must be filled, and no residual cavity should be left, and fixed to the surrounding cyst wall

Electrocoagulation hook was put into the main operation hole to make a small incision at the weakest part of the cyst and suck out the cyst fluid, and the color of the cystic fluid was observed (clear, bile-like or bloody) in the meantime (Fig. 1 A). The cyst wall was lifted and excised with an ultrasonic scalpel, with the resection range reaching more than 1/2 of the diameter of the cysts as far as possible, and the gap wall of multilocular cyst was also resected, bipolar electrocoagulation was used to stop bleeding on the cross section of the hepatic cyst wall (Fig. 1B).

#### Dissociation of pedicled greater omentum

The abdominal cavity and greater omentum were examined for adhesion and lymph node enlargement. The greater omentum was lifted by non-invasive forceps to maintain mild tension, and the greater omentum was dissociated by ultrasound knife close to the upper transverse colon without vascular area. The greater omentum dissociation of the left lobe of liver cyst starts from the left spleen and stomach ligament and ends at the right side to the pylorus region. The greater omentum of the cyst in the right lobe of the liver was dissected from the right hepatic flexure of the colon to the left. The greater omentum with pedicle was free and extended, and the gastroepiploic vascular arch must be preserved during the operation to ensure the blood supply of the free greater omentum. Dissociate the greater omentum according to the required length (about 10–15 cm) to ensure that there is no obvious tension after the greater omentum fills the hepatic cyst cavity.

#### Fixation of pedicled greater omentum

According to the preoperative location and intraoperative observation of the cyst, the pedicled greater omentum flap was introduced into the cyst cavity from the liver incision, the greater omentum and liver were fixed with 3 − 0 absorbable sutures, the middle and upper part of the greater omentum flap was fixed to the opening edge of the cyst wall, and then the top of the omental valve was placed at the bottom of the cyst. The distal end was placed in the lowest position of the cyst cavity. Considering the exudation of the cyst cavity and the liquefaction of the omentum, the filling degree was at least 80% to avoid the formation of residual cavity. Note that the free omentum can not be twisted or lumped. The greater omentum was fixed with 3 − 0 absorbable sutures around the incision of the hepatic cyst wall. Try to smooth the greater omentum and keep it in a state of no tension. Finally, the pulsatility of the fixed greater omental flap vessels was examined. Patients with concomitant gallstones underwent cholecystectomy simultaneously. Latex drainage tube was placed in the hepatic and renal crypts, and the tube was removed within 48 h after operation without the outflow of cyst fluid, bile and blood. Operative procedures are shown in Fig. 1.

#### Follow up

Follow up the patients with ultrasound and (or) CT examination at 6 and 12 months after operation, and record the results. The primary outcome measures of the study were postoperative recurrence rate and relapse-free time until the latest imaging examination and telephone follow-up.

### Statistical analysis

All calculations were statistically analyzed using SPSS for Windows version 21.0. Categorical variables are reported as counts and percentages. Measurement data were recorded as ($\stackrel{-}{x}$± s) and analyzed by analysis of variance, and enumeration data were analyzed by x^2^ test. P value < 0.05 was considered statistically significant.

## Results

### Patient characteristics

A total of 56 patients were enrolled, including 20 males and 36 females, with an average age of 65. 3 years (range, 48 to 85 years). There were 21 patients in non-omental group and 35 patients in omental group. The majority patient characteristic information is shown in Table 1.

**Table 1:** Patient characteristics information

	Omental group N (%) or ($\stackrel{-}{x}$± s)	Non-Omental group N (%) or ($\stackrel{-}{x}$± s)	F	P
Gender(male/femal)	15/20	5/16		
Age (years)	66.90 ± 11.18	66.38 ± 9.53	0.049	0.826
ASA classification				
I	28(80.0%)	18(85.7%)		
II	5(14.3%)	2(9.5%)		
III	2(5.7%)	1(4.8%)		
Symptom				
Abdominal pain	10(28.6%)	6(28.6%)		
Bloating	10(28.6%)	4(19.0%)		
Nausea	2(5.7%)	3(14.3%)		
Fever	1(2.8%)	1(4.8%)		
Weight loss	1(2.8%)	1(4.8%)		
Dysphagia	1(2.8%)	1(4.8%)		
Chest tightness	1(2.8%)	0		
No symptoms	9(25.7)	5(23.8%)		
Co-morbidities				
Hypertension	7(20.0%)	3(14.3%)		
Adiposis heptica	5(14.3%)	4(19.0%)		
Cholelithiasis	3(8.6%)	4(19.0%)		
Coronary heart disease	4(11.4%)	1(4.8%)		
Renal cyst	4(11.4%)	2(9.5%)		
Vertigo syndrome	1(2.9%)	0		
Cerebral embolism	1(2.9%)	0		
Hiatus hernia	1(2.9%)	0		
Chronic obstructive pulmonary disease	1(2.9%)	0		

### Preoperative evaluation

The diameter of the cysts ranged from 6 to 20 cm. Preoperative laboratory values between omental group and non-omental group showed no statistical difference (P > 0.05). Preoperative evaluation of patients is shown in Table 2.

**Table 2:** Preoperative information

	Omental group N (%) or ($\stackrel{-}{\varvec{x}}$± s)		Non-omental group N (%) or ($\stackrel{-}{\varvec{x}}$± s)	F	P
Largest cyst diameter (cm)		10.62 ± 3.32	10.89 ± 3.45	0.782	0.377
Single hepatic cyst		15(42.9%)	11(52.4%)		
Multiple hepatic cyst		20(57.1%)	10(47.6%)		
Predominant hepatic lobe involvement					
Left		10(28.6%)	6(28.6%)		
Right		19(54.3%)	12(57.1%)		
Bilobar		6(17.1%)	3(14.3%)		
Preoperative laboratory values					
Alanine transaminase (U/L)		35.41 ± 23.80	33.35 ± 22.36	0.291	0.592
Aspartate transaminase (U/L)		36.00 ± 19.81	33.33 ± 21.12	0.524	0.472
Total bilirubin(µmol/L)		13.91 ± 5.36	12.45 ± 4.27	1.131	0.292
Alkaline phosphatase(U/L)		82.31 ± 19.10	73.10 ± 26.29	2.296	0.136
γ-Glutamyl transpeptidase(U/L)		39.06 ± 24.12	34.62 ± 33.60	0.330	0.568
Albumin(g/L)		42.41 ± 3.33	41.72 ± 3.71	0.526	0.472

### Postoperative

**Fig. 2:**
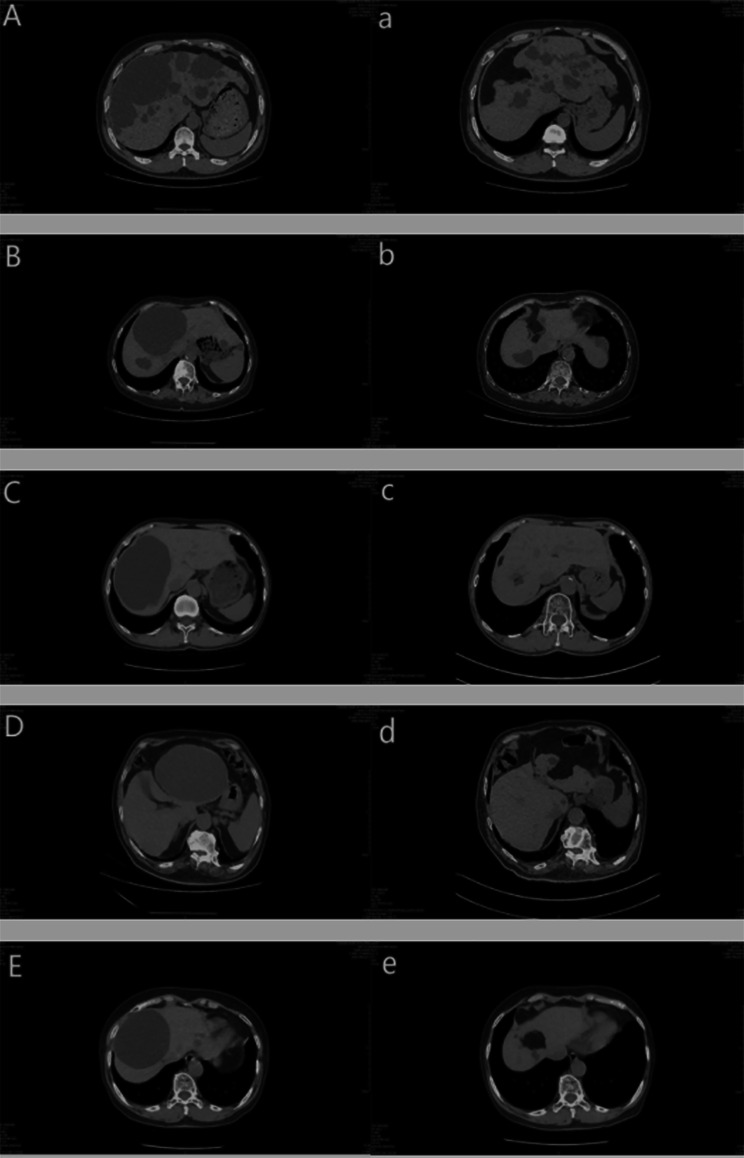
Imaging comparison between preoperative and 12 months after surgery **A,B,C,D,E** Location and size of hepatic cysts revealed by preoperative liver CT imaging **a,b,c,d,e** In the liver CT image 12 months after fenestrating hepatic cyst combined with pedicled greater omentum flap tamponade, it was found that the old hepatic cyst cavity was completely filled with pedicled greater omentum flap, and the greater omentum flap was completely anastomosed with the wall of hepatic cyst, and there was no recurrence of hepatic cyst in the operative area

**Fig. 3:**
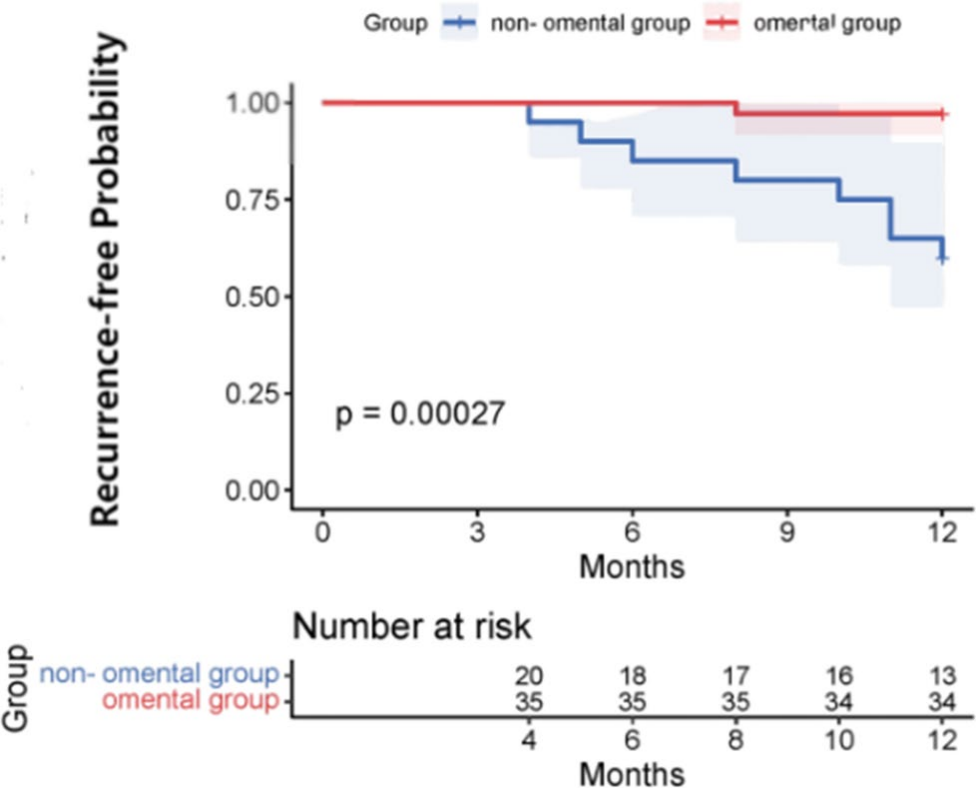
Kaplan-Meier of recurrence-free probability The recurrence at 12 months was significantly lower in the omentum group than in the non-omentum group (P=0.00011)

The postoperative laboratory values in the omental group were slightly higher than those of the non-omental group, and the albumin after the omental group was slightly lower than that of the non-omental group, but they were not statistically significant (P >0.05). Patients in the omental group had a shorter hospital stay than patients in the non-omental group (P = 0.034). The mean follow-up period was 16 months (range, 12 to 30 months). In the omental group, there was no recurrence 6 months after operation, and 1 cases (2.86%) recurred 12 months after operation. In the non-omental group 3 cases (14.28%) recurred 6 months after operation, and recurrence occurred in 8 patients (38.10%) 12 months after surgery. The recurrence rate of omental group at 6 months and 12 months after operation was significantly lower than that of non-omental group (P<0.01) (Fig. 2). In the omental group, CT images 12 months after operation showed that the cyst cavity of the primary lesion was completely filled with omentum without obvious signs of recurrence (Fig. 3). The postoperative evaluation and follow-up are shown in Table 3.

**Table 3:** Postoperative and follow-up

	Omental group N (%) or ($\stackrel{-}{x}$± s)	Non-omental group N (%) or ($\stackrel{-}{x}$± s)	F	P	***X*** ^***2***^
Postoperative laboratory values					
Alanine transaminase (U/L)	41.49 ± 43.62	33.57 ± 23.72	0.585	0.448	
Aspartate transaminase (U/L)	36.26 ± 36.23	30.90 ± 21.51	0.346	0.559	
Total bilirubin(µmol/L)	13.93 ± 5.49	13.60 ± 4.42	0.053	0.818	
Alkaline phosphatase(U/L)	82.81 ± 19.10	73.10 ± 26.29	2.296	0.136	
γ-Glutamyl transpeptidase(U/L)	32.57 ± 24.11	28.67 ± 27.57	0.309	0.581	
Albumin(g/L)	34.30 ± 3.21	35.49 ± 3.50	1.664	0.203	
Clavien-Dindo grade complication occurrence					
I	1(2.86%)	3(14.2%)			
II	0	1(4.8%)			
Postoperative complications					
Perihepatic effusion	1(2.9%)	1(4.8%)			
Ascites	0	1(4.8%)			
Fever	0	1(4.8%)			
Symptom resolution	37(94.87%)	25(96.15%)			
Operation time (min)	78.31 ± 17.36	59.05 ± 15.78	17.272	1.164E-4	
Hospitalization time (day)	5.71 ± 3.73	8.30 ± 5.01	4.751	0.034	
Hospitalization Expenses (RMB)	17236.97 ± 4645.39	17970.39 ± 5928.06	0.259	0.613	
Recurrence 6 months after surgery	0(0.00%)	3(14.28%)		0.022	5.283
Recurrence 12 months after surgery	1(2.86%)	8(38.10%)		5.089E-4	12.083

## Discussion

This study retrospectively analyzed the efficacy of laparoscopic fenestration of hepatic cysts combined with pedicled omental tamponade in the treatment of diaphragmatic hepatic cysts in a tertiary hepatobiliary surgery center. All procedures in this study were performed with a team of laparoscopic professionals, indicating that the specific location of hepatic cysts is not an absolute contraindication with skilled laparoscopic techniques. Our choice of surgery for patients with diaphragmatic hepatic cysts is based on the presence or absence of symptoms, the difficulty of excluding biliary cystadenoma and biliary cystadenocarcinoma, and the size of hepatic cysts in asymptomatic patients. Hepatic cysts on the diaphragmatic surface are located under the transverse septum and may have earlier compression symptoms, leading to diaphragmatic bulging, compression of lung tissue and esophagus. We found that many diaphragmatic hepatic cysts > 9 cm in diameter were symptomatic, while hepatic cysts in other locations were more than 15 cm in diameter. Based on our experience, we recommend early surgical treatment in asymptomatic patients with diaphragmatic hepatic cysts > 15 cm in diameter, especially in elderly patients, to avoid compression of the diaphragm by hepatic cysts and damage to lung tissue. This is consistent with the conclusion of Gomez [6].

Due to individual differences, some patients have short omentum, congenital adhesion with the abdominal wall and other factors, the tension of the diaphragm surface of the liver is large, we use an ultrasonic scalpel to separate a 10 ~ 15 cm greater omentum flap with vascular pedicle to ensure that there is no tension at the bottom of the cyst. And is fixed at the edge of the opening of the cyst to ensure the filling and drainage of the cyst. There was 1 cases of recurrence in the omentum group, and we considered that the omentum slipped due to avulsion caused by poor fixation of the omentum during the operation, so it was necessary to separate a long enough omentum, and at the same time, it was necessary to ensure that the omentum was fixed well, in order to effectively ensure that there was no recurrence. There was no postoperative intestinal adhesion, hemorrhage or necrosis of the omental flap in the omental group. Studies have shown that the recurrence rate of hepatic cysts with gallstones is increased [16]. A total of 7 patients had gallstones, and we performed cholecystectomy at the same time as liver surgery.

In this study, one patient in the omental group had a perihepatic effusion, two patients in the non-omental group had a perihepatic effusion, one patient had a small amount of ascites, and one patient had fever, which was found to be infected with a hepatic cyst during the operation. One patient had perihepatic effusion after operation, and it was the patient who had recurrence. It was considered that the packed greater omentum fell off. Therefore, we believe that the effective greater omentum flap filling the residual cavity of hepatic cyst can prevent perihepatic effusion and ascites after fenestration of hepatic cyst. The overall postoperative complication rate was 2.86% in the omental group and 14.28% in the non-omental group. There were no complications such as liver parenchyma injury, bile leakage and hemorrhage in both groups. This indicates that the omental group has fewer complications after operation, which is closely related to the absorption, immunity and regeneration of the pedicled greater omentum.

We found that the postoperative laboratory values in the omental group were slightly higher than those in the non-omental group, and the albumin in the omental group was slightly lower than that in the non-omental group, although there was no statistical significance. This may be related to the longer operation time and the anastomosis of the greater omental vessels with the hepatic cyst wall and the absorption of the cyst fluid after the pedicled greater omental flap was packed. The length of hospital stay and hospitalization cost in omentum group were shorter than those in non-omentum group. In this study, 1 patient (2.86%) in the omental group and 8 patients 8 (38.10%) in the non-omental group relapsed within 12 months after surgery, and 3 of them relapsed within 6 months after surgery (14.28%). The actual recurrence rate may be lower than the above data because the recurrent cases usually return to the hospital for review and have a higher probability of follow-up, while the cases lost to follow-up are usually those who do not recur.

This is a retrospective study. Due to the limitation of medical referral, many patients come from remote areas. Postoperative follow-up is usually a review before leaving the hospital. If there are no pathological abnormalities or recurrent symptoms, patients will not be reviewed again. Secondly, the follow-up time is relatively short, the recurrence of liver cysts can not be monitored for a long time, and the recurrence of asymptomatic patients can not be accurately assessed. More rigorous follow-up programs and more standard imaging surveillance are still needed to improve the study.

## Conclusion

The pedicled greater omentum flap tamponade combined with laparoscopic fenestration is an effective method to prevent the recurrence of diaphragmatic hepatic cysts.

## Electronic supplementary material

Below is the link to the electronic supplementary material.

Supplementary Material 1: Video

## Data Availability

All data generated or analyzed during this study are included in this published article. Further information can be obtained from the corresponding author.
